# Amyloid-β peptide signature associated with cerebral amyloid angiopathy in familial Alzheimer’s disease with *APP*dup and Down syndrome

**DOI:** 10.1007/s00401-024-02756-4

**Published:** 2024-07-18

**Authors:** Amal Kasri, Elena Camporesi, Eleni Gkanatsiou, Susana Boluda, Gunnar Brinkmalm, Lev Stimmer, Junyue Ge, Jörg Hanrieder, Nicolas Villain, Charles Duyckaerts, Yannick Vermeiren, Sarah E. Pape, Gaël Nicolas, Annie Laquerrière, Peter Paul De Deyn, David Wallon, Kaj Blennow, Andre Strydom, Henrik Zetterberg, Marie-Claude Potier

**Affiliations:** 1 Sorbonne Université, Institut du Cerveau - Paris Brain Institute - ICM, CNRS, APHP, Hôpital de La Pitié Salpêtrière, InsermParis, France; 2 Institute of Neuroscience and Physiology, The Sahlgrenska Academy at the University of Gothenburg, Gothenburg, Sweden; 3 Clinical Neurochemistry Laboratory, Sahlgrenska University Hospital, Mölndal, Sweden; 4 Department of Neuropathology Raymond Escourolle, AP-HP, Pitié-Salpêtrière University Hospital, Paris, France; 5 Department of Molecular Neuroscience, UCL Institute of Neurology, Queen Square, London, UK; 6 Department of Biomedical Sciences, Neurochemistry and Behavior, Institute Born-Bunge, University of Antwerp, Antwerp, Belgium; 7 Division of Human Nutrition and Health, Chair Group Nutritional Biology, Wageningen University and Research (WUR), Wageningen, The Netherlands; 8 Institute of Psychology and Neuroscience, King’s College London, 16 De Crespigny Park, London, UK; 9 Department of Genetics, CNRMAJ, Univ Rouen Normandie, Normandie Univ, Inserm U1245 and CHU Rouen, F–76000 Rouen, France; 10 Department of Pathology, Univ Rouen Normandie, Normandie Univ, Inserm U1245 and CHU Rouen, F–76000 Rouen, France; 11 Department of Neurology and Alzheimer Center, University of Groningen, University Medical Center Groningen (UMCG), Groningen, The Netherlands; 12 Department of Neurology, CNRMAJ, Univ Rouen Normandie, Normandie Univ, Inserm U1245 and CHU Rouen, 76000 Rouen, France; 13 Neurodegenerative Disorder Research Center, Division of Life Sciences and Medicine, Department of Neurology, Institute On Aging and Brain Disorders, University of Science and Technology of China and First Affiliated Hospital of USTC, Hefei, People’s Republic of China; 14 UK Dementia Research Institute at UCL, London, UK; 15 Hong Kong Center for Neurodegenerative Diseases, Clear Water Bay, Hong Kong, China; 16 Wisconsin Alzheimer’s Disease Research Center, School of Medicine and Public Health, University of Wisconsin, University of Wisconsin-Madison, Madison, WI USA

**Keywords:** Alzheimer’s disease, Down syndrome, Aβ peptides, Cerebral amyloid angiopathy, Mass spectrometry, Neuropathology

## Abstract

Alzheimer’s disease (AD) is characterized by extracellular amyloid plaques containing amyloid-β (Aβ) peptides, intraneuronal neurofibrillary tangles, extracellular neuropil threads, and dystrophic neurites surrounding plaques composed of hyperphosphorylated tau protein (pTau). Aβ can also deposit in blood vessel walls leading to cerebral amyloid angiopathy (CAA). While amyloid plaques in AD brains are constant, CAA varies among cases. The study focuses on differences observed between rare and poorly studied patient groups with *APP* duplications (*APP*dup) and Down syndrome (DS) reported to have higher frequencies of elevated CAA levels in comparison to sporadic AD (sAD), most of *APP* mutations, and controls. We compared Aβ and tau pathologies in *postmortem* brain tissues across cases and Aβ peptides using mass spectrometry (MS). We further characterized the spatial distribution of Aβ peptides with MS-brain imaging. While intraparenchymal Aβ deposits were numerous in sAD, DS with AD (DS-AD) and AD with *APP* mutations, these were less abundant in *APP*dup. On the contrary, Aβ deposits in the blood vessels were abundant in *APP*dup and DS-AD while only *APP*dup cases displayed high Aβ deposits in capillaries. Investigation of Aβ peptide profiles showed a specific increase in Aβx-37, Aβx-38 and Aβx-40 but not Aβx-42 in *APP*dup cases and to a lower extent in DS-AD cases. Interestingly, N-truncated Aβ2-x peptides were particularly increased in *APP*dup compared to all other groups. This result was confirmed by MS-imaging of leptomeningeal and parenchymal vessels from an *APP*dup case, suggesting that CAA is associated with accumulation of shorter Aβ peptides truncated both at N- and C-termini in blood vessels. Altogether, this study identified striking differences in the localization and composition of Aβ deposits between AD cases, particularly *APP*dup and DS-AD, both carrying three genomic copies of the *APP* gene. Detection of specific Aβ peptides in CSF or plasma of these patients could improve the diagnosis of CAA and their inclusion in anti-amyloid immunotherapy treatments.

**Supplementary Information:**

The online version contains supplementary material available at 10.1007/s00401-024-02756-4.

## Introduction

Alzheimer's disease (AD) is an age-related progressive neurodegenerative disorder characterized by the presence of amyloid plaques consisting of aggregated amyloid beta (Aβ) peptides together with neurofibrillary tangles (NFTs), neuropil threads and dystrophic neurites composed of abnormally phosphorylated tau protein. The etiology of AD is complex (multifactorial) in the vast majority of AD cases (sAD), while less than 1% of cases have a monogenic origin (ADAD, autosomal dominant AD)[52]. ADAD cases carry pathogenic variants either in the amyloid precursor protein gene (*APP)* or in the presenilin-1 (*PSEN1)* or -2 (*PSEN2)* genes or have a duplication of the *APP* gene. ADAD, AD in Down syndrome (DS-AD) and sAD, present with similar pathological features and clinical symptoms but age of onset and decease are decades earlier in ADAD and AD-DS [7, 31, 56]. Moreover, sAD and ADAD cases differ in terms of type and extent of Aβ deposits, for example a mix of diffuse and core plaques burden in sAD in contrast to predominant diffuse plaques in ADAD [14], reflecting heterogeneity of Aβ peptides composition [53]. Even among ADAD cases, various pathogenic variants result in substantial biochemical variability of Aβ peptides produced in the brain [15, 50, 82]. Together with Aβ accumulation in the parenchyma, pathologic Aβ deposits can be found in the walls of cerebral arteries and arterioles [79], and less often, capillaries and veins [62], leading to cerebral amyloid angiopathy (CAA). CAA is a type of microvascular pathology closely associated with the pathological hallmarks of AD. Indeed, moderate to severe CAA is present in 48% of sAD patients but is also found in 25% of cognitively unimpaired elderly subjects [40]. Clinically, CAA may manifest as spontaneous intracerebral hemorrhages, micro-infarcts, subarachnoid hemorrhage, transient focal neurological episodes, cognitive impairment, or dementia [13, 29, 44, 71]. CAA can occur either sporadically or in hereditary forms caused by missense pathogenic variants either in the *APP* gene, such as the Dutch-type CAA [36, 45] and in cases with Flemish and Iowa pathogenic variants in *APP* or some pathogenic variants in *PSEN1* and *PSEN2* [61], most of the time in parallel to AD. Notably, *APP* pathogenic variants leading to CAA and/or AD are localized in the juxtamembrane region of the APP protein, within the Aβ sequence. However, *APP* pathogenic variants in the transmembrane region show less prominent CAA, similarly to the *APP* London pathogenic variant [51]. Beside *APP* pathogenic variants, rare cases with *APP* locus duplication (*APP*dup) exist. These cases carry an extra copy of the *APP* locus located on chromosome 21 which results in brain APP overproduction leading to both AD and CAA. Different families have been reported with *APP* duplications, such as French [10, 27, 30, 64], British [52], Dutch [70], Finnish [6], Swedish [75], Spanish [48], and Japanese [41] kindreds. CAA pathology is also present in individuals with DS, at a higher rate as compared to sAD [11, 12, 34, 35, 51]. DS individuals carry an extra copy of human chromosome 21 and therefore overexpress APP, which explains the high prevalence of AD pathology early in life in these individuals [49, 83]. However, CAA is more pronounced in *APP*dup in comparison with DS [51], as well as CAA-associated symptoms, including hemorrhages.

All these different pathologies have the accumulation of Aβ peptides in common. Some of these peptides are important disease biomarkers and can be detected in biofluids allowing for disease diagnosis and prognosis. The Aβ42/40 ratio is an established cerebrospinal fluid (CSF) biomarker of AD pathology, for an overview see [39]. When measured in CSF of AD patients, the Aβ42 level and Aβ42/Aβ40 ratio are decreased consequently to the accumulation of these peptides in brain deposits, as assessed by either amyloid PET or at neuropathology [5]. Early studies of amyloid fibrils extracted from brains with CAA showed that CAA deposits are mainly composed of Aβ peptides with a large prevalence of Aβ40 [26]. When measured in CSF of patients with CAA, the levels of Aβ40 and Aβ42 were both found to be decreased [19, 78]. Although Aβ40 and Aβ42 peptides are generally the most abundant, other peptides such as Aβ34, Aβ37, Aβ38 and Aβ43 have also been recently recognized as having a role in modulation of AD onset and progression or as possible biomarkers for disease-associated APP and Aβ dyshomeostasis [3, 8, 18, 46, 57]. Moreover, these peptides refer to various cleavages at the C-terminal end of the longest Aβ peptides produced (Aβ48 and Aβ49). In addition, variations at the N-terminus occur, giving rise to Aβx–38, Aβx–40 and Aβx–42 [21, 63, 65]. Characterization of these different peptides is highly relevant to better understand the formation and deposition of Aβ. Indeed, various Aβ species show different aggregation, deposition and degradation properties [20], with generally longer peptides being more prone to aggregation [53, 77]. Moreover, studying the different Aβ species might lead to a better understanding and refinement of disease-specific biomarkers of Aβ-related pathologies [42].

Overall, this study aims to characterize the differences in neuropathological and brain Aβ species among *postmortem* brain samples from individuals with mild to severe CAA carrying three genomic copies of the non-mutated *APP* gene (DS with and without AD and APPdup) compared to sAD and two *APP* mutations at codon 717 which do not lead to severe CAA. We hypothesize that potential differences between conditions with APP overexpression (*APP*dup and DS) should lead to a better understanding of the neuropathological and clinical features associated with CAA and guide biomarker investigations. Several state-of-the-art techniques such as mass spectrometry (MS) and imaging-MS are applied to extensively study a cohort of pathologically confirmed brain tissues from various European brain banks, some with rare genetic conditions such as *APP*dup.

## Materials and methods

### Cases used in the study

*Postmortem* brain material from 51 individuals, categorized according to their pathological condition and genetic status were used in this study. Frozen tissue and paraffin-embedded sections were obtained from the frontal cortex (Brodmann area (BA) 9/10 regions) and hippocampus (containing CA1 and CA3 regions). Samples were provided from six European brain banks: National Brain Bank Neuro-CEB, Pitié-Salpêtrière Hospital, Paris, France, Institute of Psychiatry Kings College London Brain Bank (UK), Cambridge Brain Bank (UK), Queen Square Brain Bank (UK), Neurobiobank of the Institute Born-Bunge (Belgium) and IDIBAPS brain bank in Barcelona (Spain). The cohort included 15 controls cases (Ctrl) that did not show clinical symptoms of AD, 11 samples from patients with sporadic AD (sAD), 6 samples from patients with *APP* point mutations (2 females with the *APP*V717I mutation, 2 females and 2 males with the *APP*V717L mutation), 7 samples from patients with duplications of the *APP* locus (*APP*dup), samples from 4 cases with DS without typical AD including tau and amyloid aggregates at histological examination, and 8 samples from individuals with DS with AD (DS-AD). Sample description and case demographics are shown in Table 1 (full demographic data is shown in Supplementary Table 1). All brain banks stored *postmortem* brains in 10 to 12% formalin solution for 6 to 8 weeks before paraffin embedding. We compared available *postmortem* intervals (PMIs) of 47/51 cases between groups using one-way ANOVA. We found significant difference among means (*p* < 0.0001). Tukey’s multiple comparison test showed significant differences between *APP*V717L and all other groups except DS. Cases from each group (Ctrl, sAD, *APP* mutation at V717, *APP*dup, DS and DS-AD) were provided from two to three different brain banks.

**Table 1:** Demographic characteristics of the human brain cohort used in the study

	Ctrl (*n* = 15)	sAD (*n* = 11)	DS (*n* = 4)	DS-AD (*n* = 9)	*APP*dup (*n* = 7)	*APP* mutations V717I (*n* = 2) V717L (*n* = 4)
Sex (female/male)	11/6	5/7	2/2	2/7	1/6	4/2
Age (years) mean ± SD	71.1 ± 19.1	75.9 ± 9.2	40.7 ± 5*^#^	56.6 ± 6.5*	59.3 ± 6.9	62.8 ± 5.6
*APOE* Ɛ4 (Ɛ2/Ɛ4, Ɛ3/Ɛ4, Ɛ4/Ɛ4)	1,1,0 (1 case NA)	0,8,1	0,0,0	1,2,0	0,1,0	0,0,0 (3 cases NA)

Abbreviations: Ctrl = controls, AD = Alzheimer’s disease, sAD = sporadic AD, *APP* = amyloid precursor protein, DS = Down syndrome, DS-AD = Down syndrome with AD, NA = not available. ANOVA on ranks followed by Dunn’s post hoc test (age) or contingency Chi^2^ test (sex), were used to test differences between the groups. (*) Significant differences compared to sAD, (^#^) Significant difference compared to controls X^2^(3, N = 55) = 3.99, p = 0.2627

This study was approved by the ethical committee of ICM Paris Brain Institute (COMETH-ICM). Autopsies were authorized and performed according to each country’s current regulations.

### Histological and immunohistochemical analyses

Only the frontal cortex region was used for immunohistochemical (IHC) analysis. Formalin-fixed paraffin-embedded (FFPE) sections were deparaffinized, rehydrated and blocked. Primary antibodies, (mouse monoclonal anti-Aβ antibody clone 6F/3D, Agilent, 1/200) or the mouse monoclonal anti-pS202/T205Tau antibody clone AT8, ThermoFischer, 1/500) were applied. IHC was carried out with the Nexes automated station (Ventana Medical System Inc., Roche). Antigen retrieval consistent of CC1 solution (ULTRA Cell Conditioning solution 1; Ventana Medical System Inc., Roche), followed by a 32 min incubation was done for the detection of anti-tau AT8 antibody. The anti-Aβ 6F/3D immunohistochemistry was performed manually following formic acid pretreatment (98% formic acid for 5 min) overnight. The antibodies were detected with either the ultraView or OptiView Universal DAB Detection kit (Ventana Medical System Inc., Roche) according to the automated procedure described previously [74]. The kits included secondary antibodies (goat anti-mouse IgG) conjugated with horseradish peroxidase.

Hematoxylin and eosin (H&E) staining of the frontal cortex sections from sAD, *APP*dup and DS-AD cases that showed the highest CAA intensity were performed to analyze the presence of microbleeds and microhemorrhages.

### Analysis of Aβ and tau pathologies

Following IHC, sections were scanned on a Hamamatsu Nanozoomer HT2. A semi-quantitative four-grade score (i.e*.*, 0: no, 1: low, 2: moderate, 3: high) for 16 neuropathological features was done: (1) Aβ load in the parenchyma, (2) amount of coarse-grained plaques, (3) amount of Aβ deposits in the wall of arteries, arterioles and venules, (4) number of Aβ positive arteries, arterioles and venules, (5) amount of Aβ deposits in capillaries, (6) number of Aβ positive capillaries, (7) amount of Aβ in meningeal vessels, (8) number of positive meningeal vessels, (9) amount of perivascular Aβ, (10) amount of subpial Aβ aggregates, (11) amount of Aβ deposits in the white matter, (12) total load of pTau, (13) number of neuritic plaques (NP) (identified by the dystrophic neurites that surround the plaque core of the neuritic plaque), (14) number of neurofibrillary tangles, (15) amount of neuropil threads. In addition, we also analyzed the proportion of diffuse-over focal-type Aβ plaques [22] using a three-grade scale (i.e*.*, 1: diffuse deposits exceed focal deposits; 2: diffuse and focal deposits are similar; 3: focal deposits exceed diffuse deposits). Each paraffin section was examined independently by two experienced neuropathologists (SB and LS). The degree of agreement between them was quantified by kappa using GraphPad Prism as previously described [43]. The agreement ranged from moderate to almost perfect. In instances where observers did not reach a consensus, a consensus meeting was held to establish a final score.

### Protein extraction and immunoprecipitation from frozen human *postmortem* brain tissues

Based on frozen sample availability, mass spectrometry analysis was performed on a subset of samples, as indicated in the respective figures and Supplementary Table 1.

Frozen cortical and hippocampal brain tissues available (Supplementary Table 1) were homogenized and immunoprecipitated according to Gkanatsiou et al*.* [23]. Briefly, 150 mg of tissue was homogenized in (Tris)-buffered saline (TBS), pH 7.6 containing protease inhibitor (Roche), for 4 min at 30 Hz using a TissueLyzer II (Qiagen). After centrifugation (31,000×g for 1 h at 4 °C), the supernatant (TBS fraction) was aliquoted and stored at – 80 °C. The pellet was resuspended and homogenized in 1 ml of 70% (v/v) formic acid (FA). After sonication for 30 s and centrifugation at 31,000×g for 1 h at 4 °C, the supernatant (FA fraction) was dried in a vacuum centrifuge. Aβ peptides were immunoprecipitated from both fractions using two mouse monoclonal antibodies in combination: 6E10 and 4G8 (cat: 803,003 and 800,711, respectively, BioLegend). Four µg of each antibody was independently conjugated with 50 µl IgG-coated magnetic beads (Dynabeads M–280 Sheep anti-mouse, cat: 11202D, Thermo Fisher Scientific) according to manufacturer’s procedures. After conjugation with the beads, the two antibodies were combined and added to each sample. Prior to immunoprecipitation (IP), the dried FA fraction was reconstituted in 200 µL 70% FA (v/v) and shaken for 30 min at room temperature. The reconstituted samples were then centrifuged at 31,000 × g for 1 h at + 4 °C and the supernatant was moved to a new tube and neutralized with 4 mL of 0.5 M Tris buffer. TBS and FA reconstituted fractions were incubated over night at + 4 °C with antibody-conjugated beads in 0.2% Triton X–100 (w/v) solution. The day after, the samples were washed and Aβ peptides eluted in 100 µl 0.5% FA (v/v). Eluates were dried down in a vacuum centrifuge and stored at − 80 °C pending MS analysis. Samples were normalized according to sample volume (i.e., the same volume of brain extract was used for immunoprecipitation).

### Mass spectrometry analysis

Aβ peptides immunoprecipitated from TBS and FA fractions were analyzed by matrix-assisted-laser-desorption/ionization time-of-flight/time-of-flight MS (MALDI-TOF/TOF–MS) using a Bruker Daltonics UltraFleXtreme instrument as described previously [24, 58]. Prior to MS analysis, samples were reconstituted in 5 μl 0.1% FA/20% acetonitrile in water (v/v/v), shaking for 30 min at room temperature. The FlexAnalysis software 3.4 (Bruker Daltonics) was used for MALDI spectrum analysis. For each spectrum the individual Aβ peak areas were normalized to the sum of all Aβ peak areas in the same spectrum before further analysis, to obtain a relative abundance of each Aβ species and thus an Aβ pattern profile for each spectrum. The 3 μl left from MALDI preparation was dried and reconstituted in 7 μl 8% FA/8% acetonitrile in water (v/v/v), for subsequent analysis by nanoflow liquid chromatography (LC) coupled to electrospray ionization (ESI) hybrid quadrupole–orbitrap tandem MS using a Dionex Ultimate 3000 system and a Q Exactive (both Thermo Fisher Scientific), as described previously [24, 26].

### Sample preparation for MALDI-MS imaging

For MALDI MS imaging (MSI), we employed a previously validated protocol for robust peptide and protein mass spectrometry imaging [54]. Frozen tissue sections were thawed and dried under vacuum for 15 min. A series of sequential washes of 100% ethanol (60 s), 70% ethanol (30 s), Carnoy’s fluid (6:3:1 ethanol/CHCl_3_/acetic acid) (90 s), 100% ethanol (15 s), H_2_O with 0.2% TFA (60 s), and 100% ethanol (15 s) were carried out. Tissue sections were subjected to formic acid (FA) vapor for 25 min. A mixture of 2,5-dihydroxyacetophenone (2,5-DHAP) and 2,3,4,5,6-pentafluoroacetophenone (PFAP) was used as matrix compound and applied using a HTX TM-Sprayer (HTX Technologies LLC, Carrboro, NC, USA). A matrix solution of 5.7 µl/ml PFAP and 9.1 mg/ml of DHAP in 70% ACN, 2% acetic acid/2% TFA was sprayed onto the tissue sections using the following instrumental parameters: nitrogen flow (10 psi), spray temperature (75 °C), nozzle height (40 mm), seven passes with offsets and rotations, and spray velocity (1000 mm/min), and isocratic flow of 100 μl/min using 70% ACN as pushing solvent.

### MALDI MS imaging

MALDI-MSI experiments were performed on a rapifleX Tissuetyper MALDI-TOF/TOF instrument (Bruker Daltonics) using the FlexImaging software (v5.1, Bruker Daltonics). Measurements were performed at 10 μm spatial resolution, at a laser pulse frequency of 10 kHz with 200 shots collected per pixel. Data were acquired in linear positive mode in the mass range of 1500–6000 Da (mass resolution: m/Δm_FWHM_ = 1000 at m/z 4515). Prior to MSI e-acquisition calibration of the system was performed using a combination of peptide calibration standard II and protein calibration standard I, to ensure calibration over the entire mass range of potential Aβ species.

### Statistical analysis

GraphPad Prism software version 9.3.1 (GraphPad Software, La Jolla, CA, USA) was used for statistical analysis. The non-parametric one-way ANOVA on ranks (Kruskal–Wallis) followed by Dunn’s test for multiple comparison was used when comparing more than two groups. A comparison of the distribution of semi-quantitative scores of Aβ and tau immunostaining was performed using the Chi^2^-square test followed by Fisher exact between group comparisons. We performed non-parametric correlations (Spearman rho) between histological CAA scores and the most abundant LC–MS Aβ peptides among the patient sample. Considering the ABC score (Amyloid, Braak, CERAD) [37], a hybrid CAA score was created ranging from 0 to 38 aiming at reflecting CAA abundance in each individual. It included: (a) number of Aβ positive vessels (0–3), (b) Aβ quantity in the wall of arteries/arterioles/venules (0–3), (c) number of Aβ positive capillaries (0–3), (d) Aβ quantity in the wall of capillaries (0–3), (e) perivascular Aβ (0–1) and (f) subpial Aβ (0–1). A weighting of 4 was given to the spatial spreading subscores (a, c, e, f), and of 1 to the density subscores (b, d).

## Results

### Aβ deposits in the parenchyma and in blood vessels differ between sAD, DS and ADAD cases with *APP*dup and *APP* mutations

**Fig. 1:**
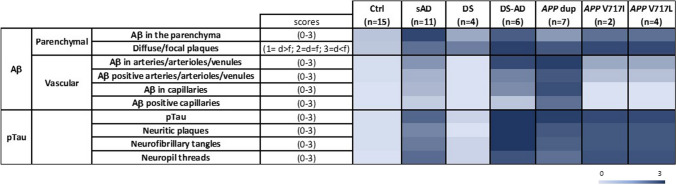
Quantification of Aβ and Tau pathologies in *postmortem* human cortex from controls, sAD, DS, DS-AD, *APP*dup and *APP* mutations. Heatmap showing mean scores for 10 AD neuropathological features across groups. sAD = sporadic AD, *APP*V717I and *APP*V717L = AD with *APP* mutations at codon 717, *APP*dup = *APP* microduplication, DS = Down syndrome, DS-AD = Down syndrome with AD. Lightest blue corresponds to score 0, and darkest blue to score 3

**Fig. 2:**
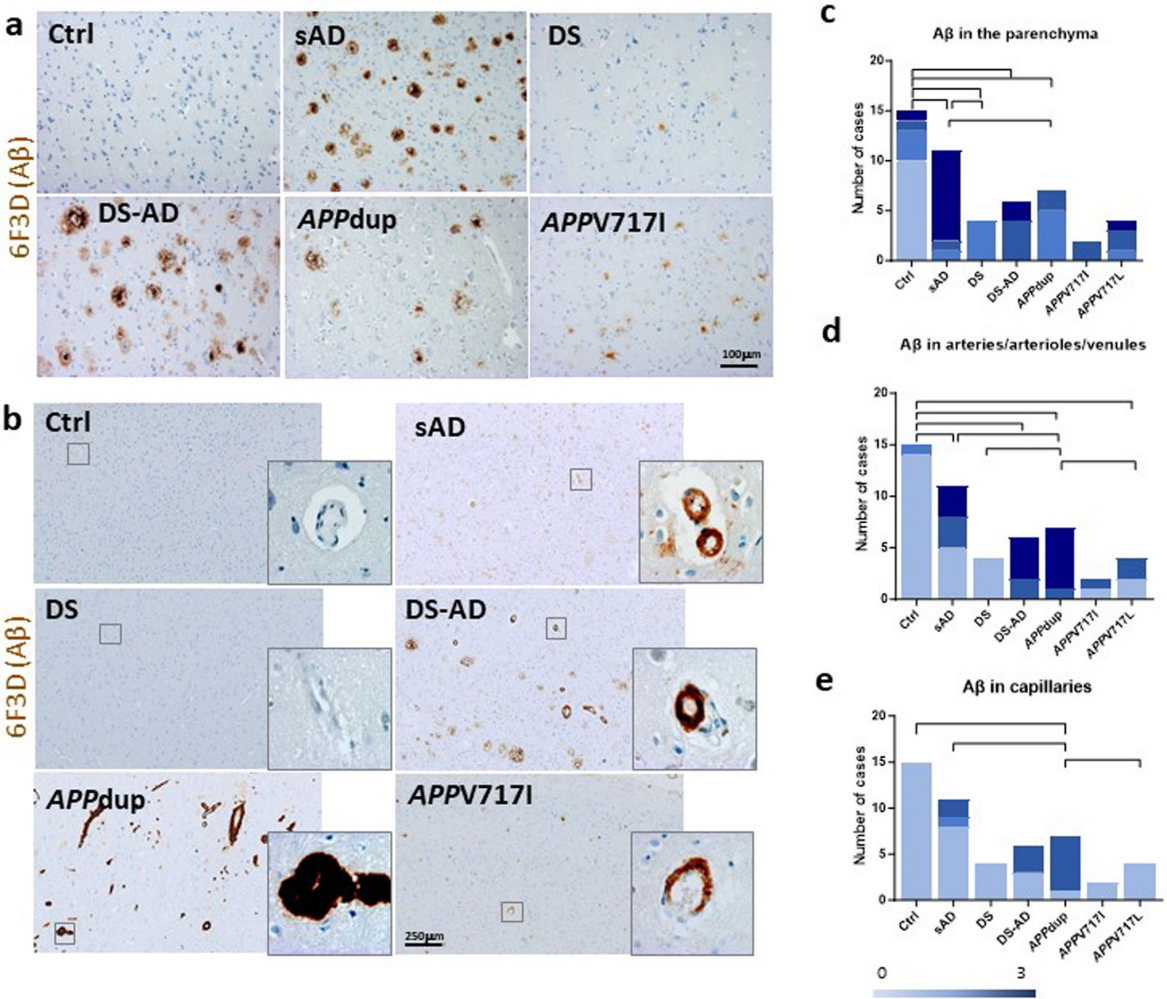
Immunohistochemical quantification of parenchymal and vascular Aβ deposits in *postmortem* human cortex from controls, sAD, DS, DS-AD, *APP*dup and *APP* mutations. Representative images of anti-Aβ 6F3D immunohistochemical staining in the parenchyma (**a**) and blood vessels (**b**) (scale bar is 100 µm in A and 250 µm in B). Distribution of the number of cases in each group with Absence = score 0, Low = score 1, Moderate = score 2, High = score 3 levels of Aβ deposits in brain parenchyma (**c**), in the wall of arteries/arterioles/venules (**d**) and capillaries (**e**). Chi^2^-Square tests, *α* = 5%, comparisons with* p*-values *p* < 0.05 considered as significant are indicated as bars. Ctrl = control, sAD = poradic AD, *APP*V717I and *APP*V717L = AD with *APP* mutations at codon 717, *APP*dup = *APP* microduplication, DS = Down syndrome, DS-AD = Down syndrome with AD

Frontal cortical areas (BA9/10 or BA9) from 49 cases (15 controls, 11 sAD, 4 DS, 6 DS cases with pathological AD (DS-AD), 7 *APP*dup, 2 ADAD with the London *APP*V717I mutation and 4 ADAD with the *APP*V717L mutation) were analyzed for Aβ deposits in the parenchyma and in the blood vessel walls (Supplementary Table 2). According to different featured detailed above in Materials and Methods, Aβ scores as well as number of cases with either parenchymal or vascular Aβ deposits differed significantly across conditions. Regarding Aβ parenchymal deposits, when considering the average load between cases within each group, we found the highest levels in sAD, DS-AD and *APP* mutations. In DS-AD and *APP*V717I, there was a predominance of focal-type Aβ deposits. In sAD and *APP*V717L the type of parenchymal deposits was variable (Figs. 1, 2a and Supplementary Table 2). Lower levels of Aβ parenchymal deposits were detected in DS cases and in *APP*dup, with mostly diffuse-type deposits in DS cases and focal-type plaques in *APP*dup (Figs. 1,  2a, and Supplementary Table 2).

**Fig. 3:**
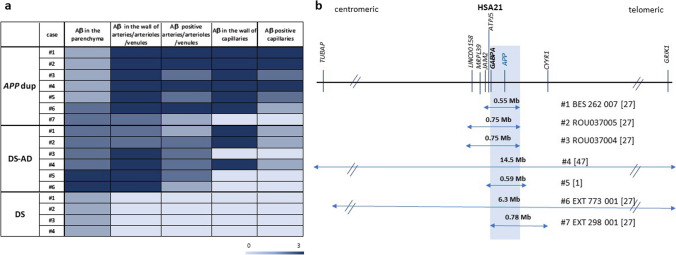
Parenchymal and vascular Aβ deposit differences between cases with *APP*dup, DS and DS-AD. (**a**). Heatmap of scores across cases revealed striking differences between *APP*dup and DS-AD. Lightest blue corresponds to score 0, and darkest blue to score 3. (**b**) Overlap of the duplicated segment from HSA21 in various cases with *APP*dup includes two genes: *APP* and *GABPA*. *APP*dup = *APP* microduplication, DS = Down syndrome, DS-AD = Down syndrome with AD

We then analyzed Aβ deposits in blood vessels (i.e., CAA) and observed that the *APP*dup and DS-AD cases had the highest number of vessels with Aβ deposits and that these deposits were abundant compared to all other groups with AD (Figs. 1, 2b, 3a). Indeed, in arteries, arterioles, and venules, all *APP*dup and DS-AD cases showed CAA. Furthermore, the highest score (3) for load of Aβ in the vessel wall was found in 4 out of 6 DS-AD cases and in 6 out of 7 *APP*dup cases (Figs. 1e,  2b,  3a). However, we observed Aβ deposits in the vessel walls only in 50% of sAD cases and, of these cases, half of them showed high levels of Aβ deposits while the other half had moderate levels of Aβ deposits (Fig. 2b and 2d). In the DS cases, Aβ deposits were not observed in arteries, arterioles, venules, or capillaries. This lack of detection could be attributed either to the limited number of cases analyzed, (only four individuals were included in the study) or to their younger age. (Figs. 1,  2b,  2d,  3a, Table 1).

We further analyzed Aβ deposits in the capillaries. Interestingly, in our series, all except one (86%) of the *APP*dup cases showed Aβ deposits in the capillaries, while only 50% of DS-AD and only 3 out of 11 (27%) sAD had Aβ deposits in the capillaries. Absence of capillary Aβ deposits was noted in Ctrl and DS, but also in *APP* mutations cases (Table 1 and Supplementary Table 2). In cases with a high number of capillaries containing Aβ deposits, the load of Aβ was high, except in one of the three sAD, in which the amount of Aβ deposits in the capillary walls was moderate. (Fig. 2e).

Other Aβ deposits, such as perivascular, subpial and white matter were analyzed. Aβ deposits around arterioles and capillaries often result in capillary occlusion, referred to previously described dysphoric CAA [2, 73]. While perivascular depositions were absent in all Ctrl, DS and DS-AD cases, they were more frequent in subjects with sAD, *APP*dup and *APP*V717 mutations. Aβ in the white matter was seen in all DS-AD and *APP*V717I cases and in the majority (82%) of the sAD subjects. Deposits were less abundant in *APP*V717L and *APP*dup cases (50% and 29% of cases, respectively). Subpial deposits were seen in all groups except in DS. These deposits were more abundant in sAD and *APP* mutations cases compared to the other groups (Supplementary Table 2). Assessing Aβ deposits in leptomeningeal vessels proved challenging due to inconsistent presence or completeness of meninges across cases, hindering systematic evaluation.

Since cases with *APP*dup showed major differences in Aβ deposition, not only in blood vessels but also in the parenchyma, we wondered whether the size of the duplicated genomic segment encompassing the *APP* gene could explain the neuropathological differences and which other genes than *APP* could possibly be involved*.* The size of the duplicated segment in the 7 cases studied here varied between 0.55 and 14.5 Mb (Fig. 3B). Two *APP*dup cases were from the same family while the other 5 were from 5 different families [1, 27, 48, 64]. In our series of samples, we found that the overlapping duplicated segment contained two genes: *APP* and *GABPA* (GA binding protein transcription factor subunit alpha).

Finally, we searched for the presence of microbleeds and/or micro hemorrhages on hematoxylin–eosin-colored cortical sections from 3 sAD, 5 *APP*dup and 6 DS-AD cases. Two *APP*dup and one DS-AD cases had rare microbleeds (hemosiderin traces around cortical vessels) and one DS-AD case had microinfarcts seen as less than 1 mm discoloured regions (Supplementary Table 2).

To summarize, we found that Aβ deposition in blood vessel walls was clearly associated with *APP*dup and DS-AD cases, and within these cases, Aβ deposition was seen in the capillaries in a higher proportion compared to DS-AD. However, it was in sAD cases where parenchymal Aβ was predominant. Finally, cases with *APP* point mutations showed a moderate phenotype in the blood vessels (arteries, arterioles, and venules), without capillary involvement.

### Tau pathology is very high in sAD, DS-AD and ADAD cases with *APP*dup and *APP* mutations

**Fig. 4:**
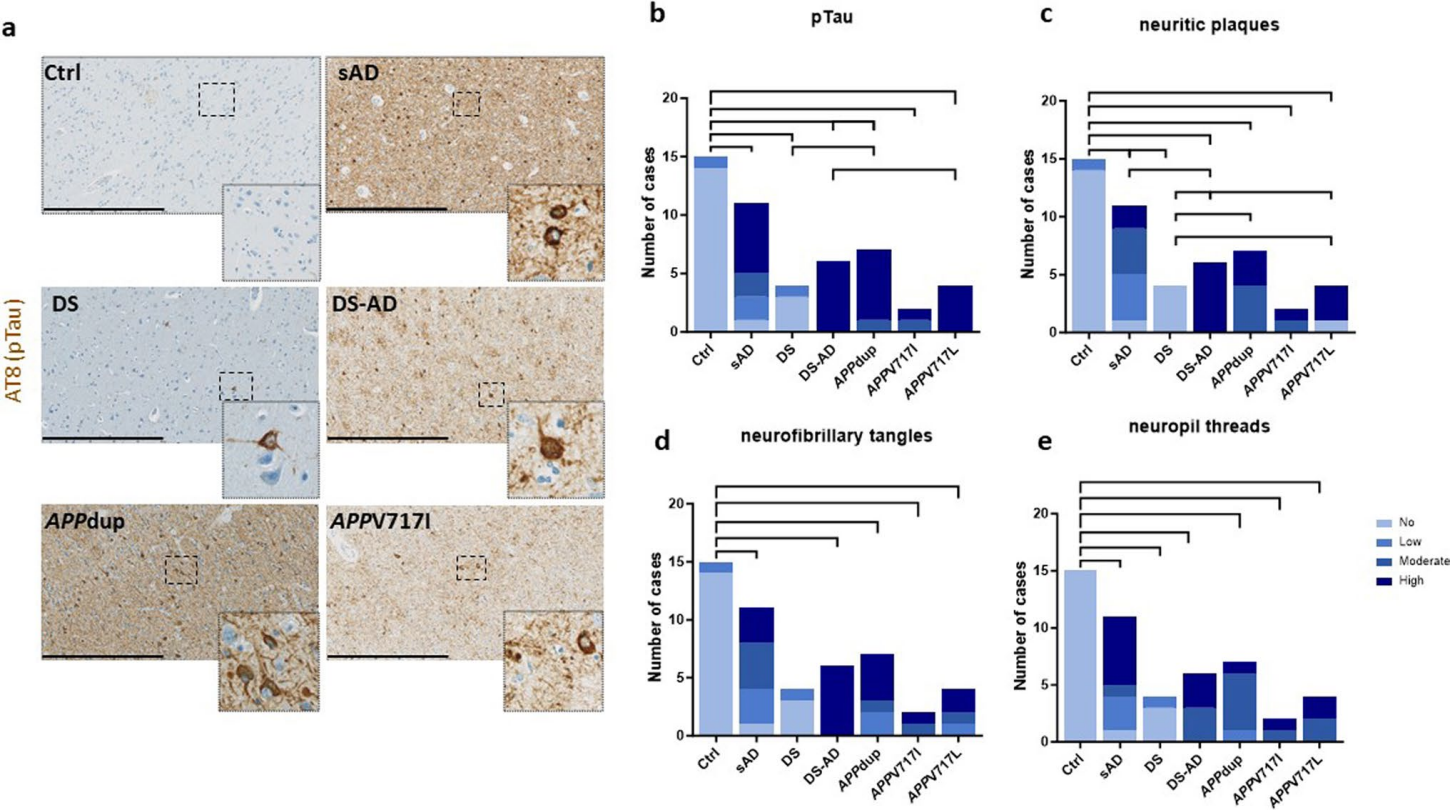
Immunohistochemical quantification of pTau pathology in *postmortem* human cortex from controls, sAD, DS, DS-AD, *APP*dup and *APP* mutations. (**a**) Representative images of anti-pTau AT8 immunohistochemistry (scale bar 800 µm). (**b**). Distribution of the number of cases in each group with No = score 0, Low = score 1, Moderate = score 2, High = score 3 levels of pTau deposits (**c**), neurofibrillary tangles (**d**) and neuritic plaques (**e**). Chi^2^ tests, *α* = 5%, comparisons with p-values p < 0.05 considered as significant are indicated. Ctrl = control, sAD = sporadic AD, *APP*V717I *and APP*V717L = AD with *APP* mutations at codon 717, *APP*dup = *APP* microduplication, DS = Down syndrome, DS-AD = Down syndrome with AD

We analyzed intracellular tau deposits by immunostaining of adjacent paraffin sections from frontal cortical areas (BA9/10 or BA9) of the 49 cases using the phosphorylation dependent anti-Tau antibody (clone AT8) (Fig. 4, Supplementary Table 2). The proportion of cases with pTau deposits were comparable across all AD-related cases with slight differences. All cases with AD except one sAD showed pTau immunoreactivity (Fig. 1 and 4a). This case (NeuroCEB5786 in Supplementary Table 1) was classified as Braak V Thal 5 with CAA in the neuropathological report provided by the Brain Bank. AT8 immunoreactivity was present in the hippocampus and entorhinal cortex as well as neocortex of the temporal lobe, including the superior temporal gyrus, and in secondary visual area in the occipital cortex. However, it was rare or absent in the frontal cortex. We selected this case among sAD cases because of the presence of CAA. There was no detectable immunoreactivity in Ctrl and DS brains except in two cases (one case per group) that showed very low level of tau pathology (Fig. 4a and b, Supplementary Table 2). All DS-AD and *APP*V717L cases had high scores of pTau. In the *APP*dup cases all had high scores except one subject that had moderate (Fig. 4b, Supplementary Table 2). In DS-AD a high load of pTau was observed in 55% of cases and in 50% of cases in *APP*V717I. Therefore, the distribution of the number of cases with various levels of pTau deposits was significantly different between controls and all groups (Fig. 4b).

When analyzing NFTs, the highest number was observed in DS-AD cases followed by *APP*dup. In these two groups, all cases presented NFTs. Even though most sAD cases had NFTs (91%), the number of NFTs was lower than in the DS-AD and *APP*dup groups. (Fig. 4d).

We further analyzed the amount of neuritic plaques and neuropil threads between pathological conditions. Distribution of the number of cases with various levels of neuritic plaques or neuropil threads was significantly different between controls and all other groups (Fig. 4c, e).

Altogether, we found that tau pathology was very high in all brain samples from patients with AD. Abundant tau pathology was associated with a high burden of parenchymal Aβ deposits in all groups with AD except the *APP*dup cases where Aβ deposits in the walls of blood vessels and capillaries predominated over parenchymal aggregates (Supplementary Table 2).

### MALDI Aβ profiling in cortex and *hippocampus* shows subtle differences between sAD, DS and ADAD cases with *APP*dup and *APP* mutations.

**Fig. 5:**
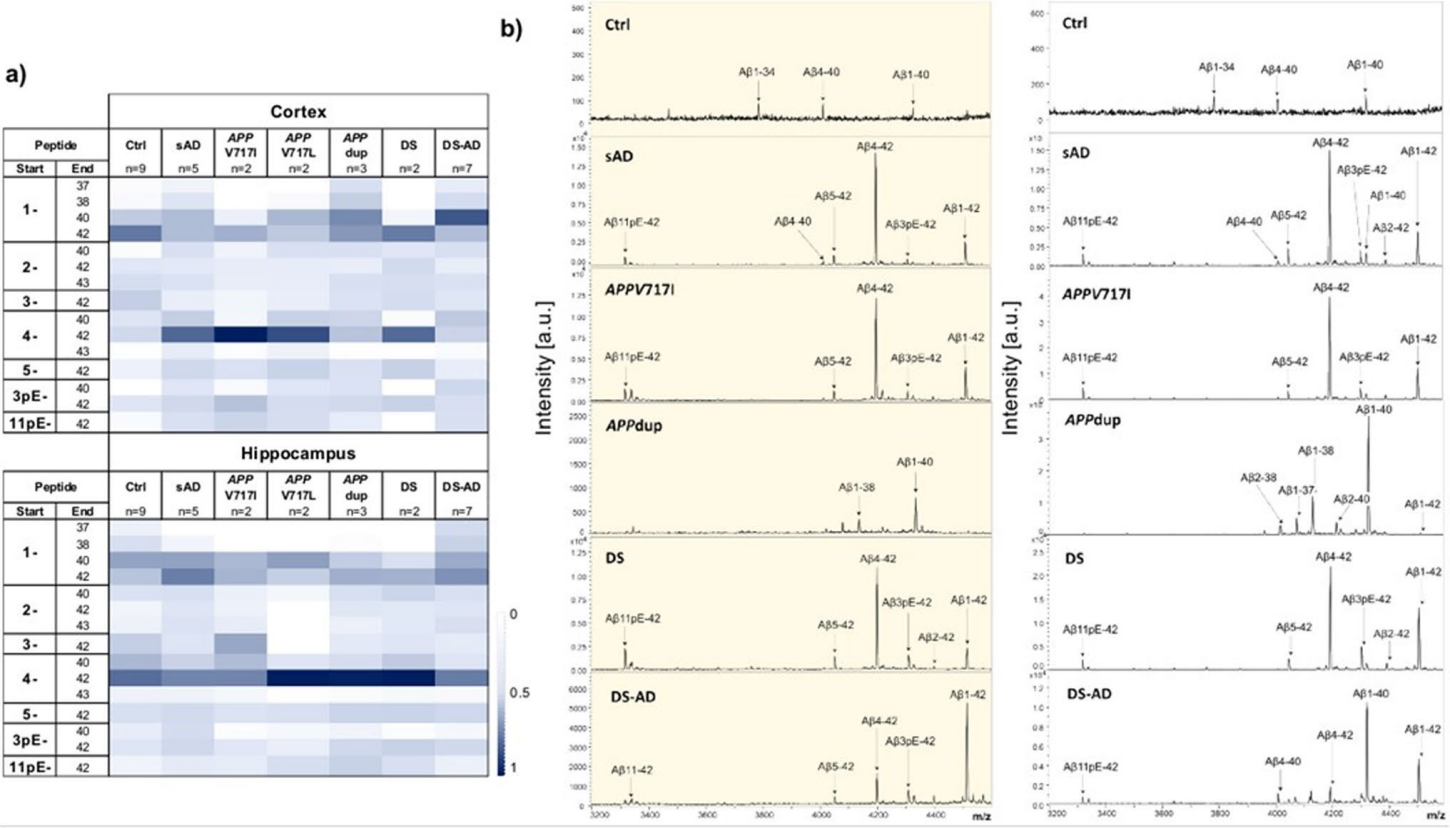
MALDI analysis showing relative abundance of Aβ peptides in the hippocampus and cortex of controls, sAD, ADAD, *APP*dup, DS and DS-AD **cases**. Tables in panel (**a**) show normalized mean areas of the most abundant Aβ peptides in the cortex and hippocampus. Panel (**b**) shows one representative mass spectra per group side by side, respectively in hippocampus (yellow panel on the left) and in the cortex (on the right). The identity of the most intense peaks are indicated with the label. Ctrl = control, sAD = sporadic AD, *APP*V717I *and APP*V717L = AD with *APP* mutations at codon 717, *APP*dup = APP microduplication, DS = Down syndrome, DS-AD = Down syndrome with AD

To assess qualitative differences in Aβ peptides composition in *postmortem* matched hippocampal and cortical samples, a subset of cases characterized neuropathologically were first analyzed with MALDI. IP on the insoluble fraction (FA) using a combination of the 6E10 and 4G8 antibodies, was used to enrich for a diversity of Aβ truncated peptides. Mean relative abundance of the detected peptides are shown in Fig. 5a and representative MALDI spectra are shown in Fig. 5b. In total, 31 Aβ peptides were detected, of which the 15 most abundant were included in further analysis. MALDI spectra were comparable in the cortex and hippocampus; the exception being DS-AD. Generally, the Aβ1–x and 4–x peptides were the most abundant. Aβ1–40 levels, together with Aβ1–37 and 1–38 were higher in *APP*dup and DS-AD groups in the cortex. Aβ4–42 was particularly high in sAD, *APP* mutations, and DS group in the cortex, while surprisingly it was also the highest in the *APP*dup group in the hippocampus. However, this high level of Aβ4-42 was driven by only one case, specifically case #5, which had also high Aβ1-42, while the other 2 cases did not show the presence of this peptide. Moreover, no significant correlation was found between the hippocampus and cortex for this peptide in *APP*dup, possibly indicating different independent processes in these two different areas (data not shown). Peptide profiles of control cases also showed Aβ signals, Aβ1-42 peptide being the most prominent in the cortex and Aβ4-42 in the hippocampus. This is not uncommon and has been previously reported in other control brains [25]. However, the presence of Aβ deposits at IHC analysis was not as spread and prominent to characterize these brains as having amyloid pathology. Note that the MALDI data reflects the *relative* amount in each sample as described in the Method section.

To get better quantitative data, samples were further analyzed by LC–MS. Due to the limited amount of hippocampal samples, only cortical samples were subjected to LC–MS.

### LC–MS analysis reveals high abundance of Aβ peptides in *APP*dup cases compared to all other groups

**Fig. 6:**
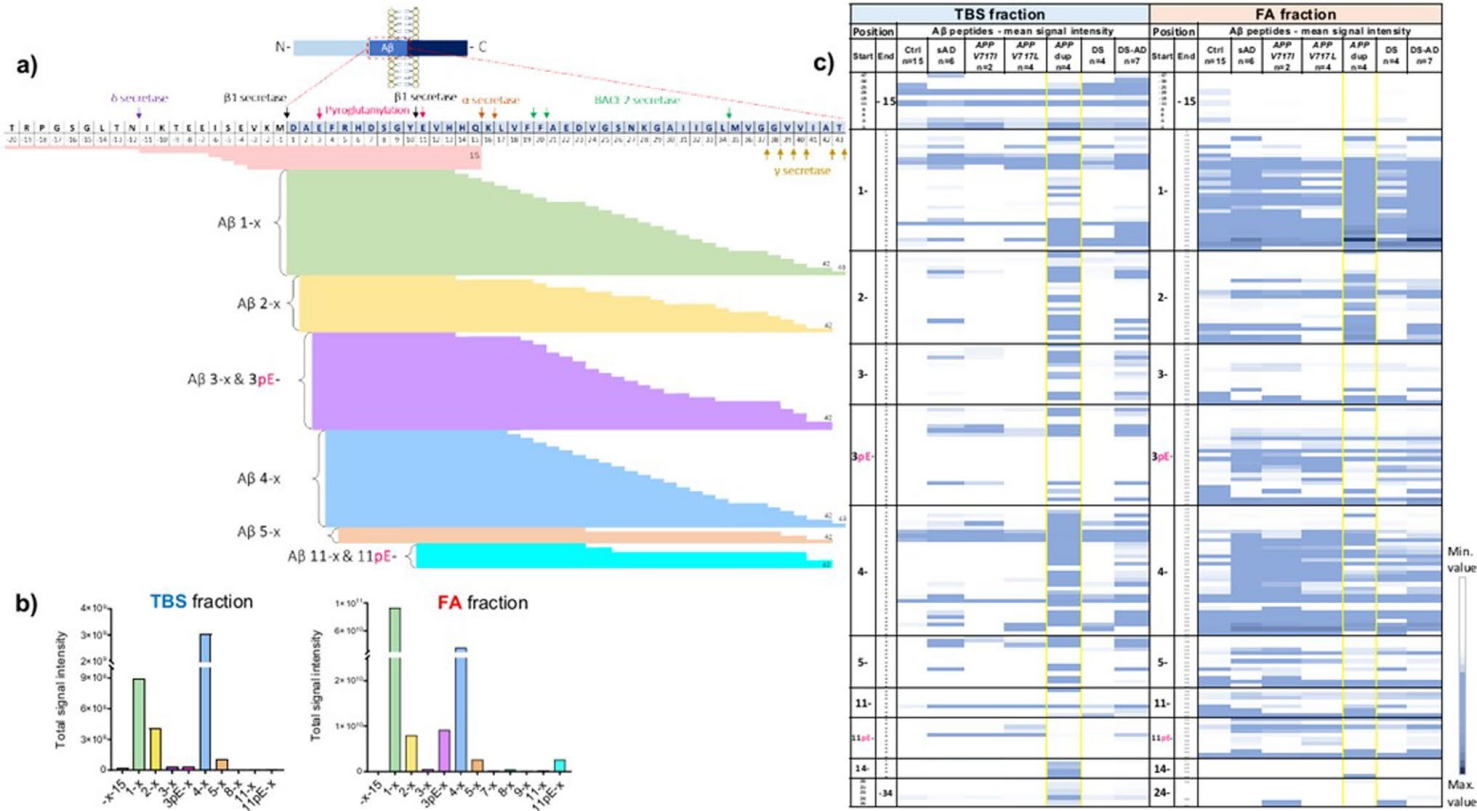
Overview of the Aβ peptides detected by LC/MS–MS analysis in the soluble and insoluble fractions. (**a**) Schematics of the Aβ peptide and possible enzymatic cleavages, followed by a graphic representation of the main peptides detected by LC/MS–MS, and (**b**) different abundance of Aβ peptide in the two fractions. Bar graphs represent the sum of the detected peptides in all groups. (**c**) Comparison of the mean abundance of the different peptides in the two fractions analyzed. Tables are color-coded based on lowest, 50 percentile and highest value. TBS fraction = soluble fraction, FA fraction = insoluble fraction, Ctrl = control, sAD = sporadic AD, *APP*V717I *and APP*V717L = AD with *APP* mutations at codon 717, *APP*dup = APP microduplication, DS = Down syndrome, DS-AD = Down syndrome with AD

**Fig. 7:**
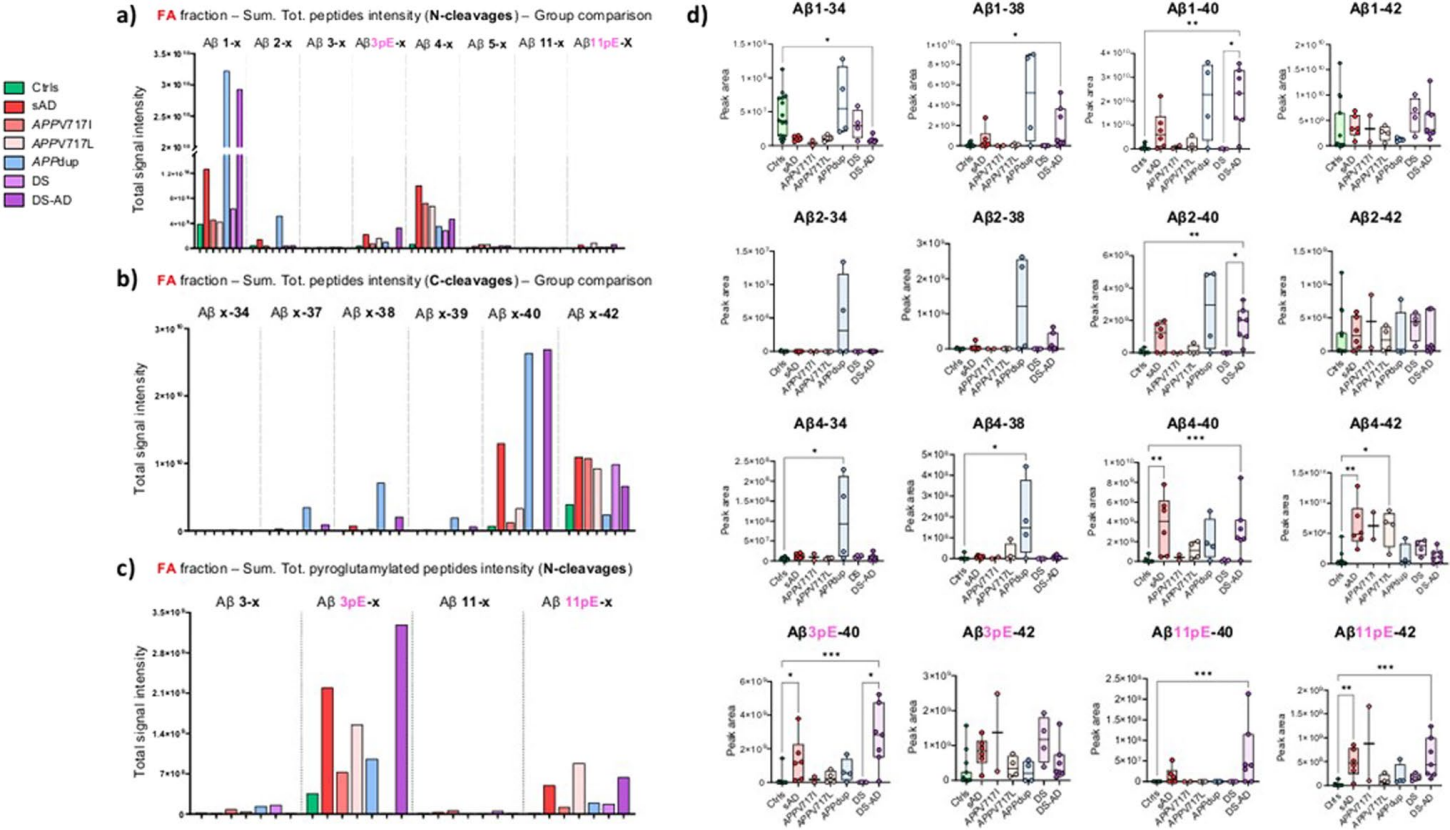
Detailed analysis of C- and N-cleavages of Aβ peptides in the insoluble FA fraction. A, b) From top to bottom, three bar-graphs representing the sum of the total intensity of different peptide-groups with the same N-terminal or C-terminal end and different cleavages. Panel (**c**) focus on pyroglutamylated Aβ forms at position 3- and 11-. (**d**) Scatter plots showing data distribution of the most abundant peptides in the different groups. P-values determined using Kruskal–Wallis followed by Dunn’s test to adjust for multiple comparison (^a^*p* < 0.033, ^b^*p* < 0.002, ^c^*p* < 0.001). Ctrl = controls, sAD = sporadic AD, *APP*V717I *and APP*V717L = AD with *APP* mutations at codon 717, *APP*dup = *APP* microduplication, DS = Down syndrome, DS-AD = Down syndrome with AD. Aβ3pE, 11pE = pyroglutamylated peptides

Using nanoflow LC–MS, we identified a total of 134 Aβ peptides in the soluble fraction (TBS) and 161 in the FA fraction, with both N- and C-terminal truncations. Not only truncated but also pyroglutamylated peptides at position -3 (3pE) and -11 (11pE) of the Aβ sequence were detected. A complete peptide list with acquisition characteristics is presented in Supplementary Table 3. Most Aβ peptides were detected in the FA fraction, which generally contains species originating from insoluble aggregates while the TBS fraction contains soluble Aβ species [16]. To fully characterize the Aβ profile, both soluble and insoluble fractions needed to be analyzed. A scheme of all Aβ peptides with possible cleaving enzymes is represented in Fig. 6a and all detected peptides together with the relative abundance of the different peptide-groups in Fig. 6b Figure 6c shows a heatmap of the mean abundance of all peptides in the different groups and fractions. In line with the MALDI data, we found that the C-terminally truncated 1–x and 4–x peptides were the most abundant, followed by truncated peptides at position 2–x, both TBS and FA fractions, and the pyroglutamylated peptides at position 3 in the FA fraction (Fig. 6b). In the TBS fraction, the *APP*dup group clearly showed the highest abundance of Aβ peptides (Fig. 6c). Peptides C-terminally truncated at 15/16 and extending N-terminally of the BACE1 cleavage site were mainly found in the TBS fraction but were almost equally present across cases and groups (Fig. 6c). A more detailed analysis of the peptide profiles in the FA fraction is shown in Fig. 7. In this fraction, the *APP*dup and DS-AD groups showed a more similar profile compared with the sAD and *APP* mutations groups. The *APP*dup and DS-AD groups showed similar amount of C-terminally cleaved Aβ peptides, mostly 1–x and 4–x (Fig. 7a, b). However, the *APP*dup group was enriched in peptides cleaved at position 2 (2–x) compared to DS-AD and all other groups (Fig. 7a, d). The *APP*dup group also showed higher abundance of N-truncated Aβ peptides ending at position 34 (x–34), together with higher Aβx–37, x–38 and x–39 compared to all other groups. The Aβx–40 peptides were the most abundant peptides, highly present in both *APP*dup and DS-AD group (Fig. 7b, d). On the contrary, the *APP*dup group displayed very low relative abundance of Aβx-42 peptides compared to all other groups. Furthermore, pyroglutamylated forms of Aβ also showed low relative abundance in this group. Differences often did not reach statistical significance due to the small sample size of the cohort (Fig. 7d).

Interestingly, sAD samples had higher amounts of Aβ peptides compared with *APP* mutations cases. The mean age difference of more than 10 years between the two groups might have led to the increased accumulation of Aβ in the brain of sAD cases.

The control group showed the lowest amount of Aβ peptides, as expected, followed by the DS group. (Fig. 7).

**Fig. 8:**
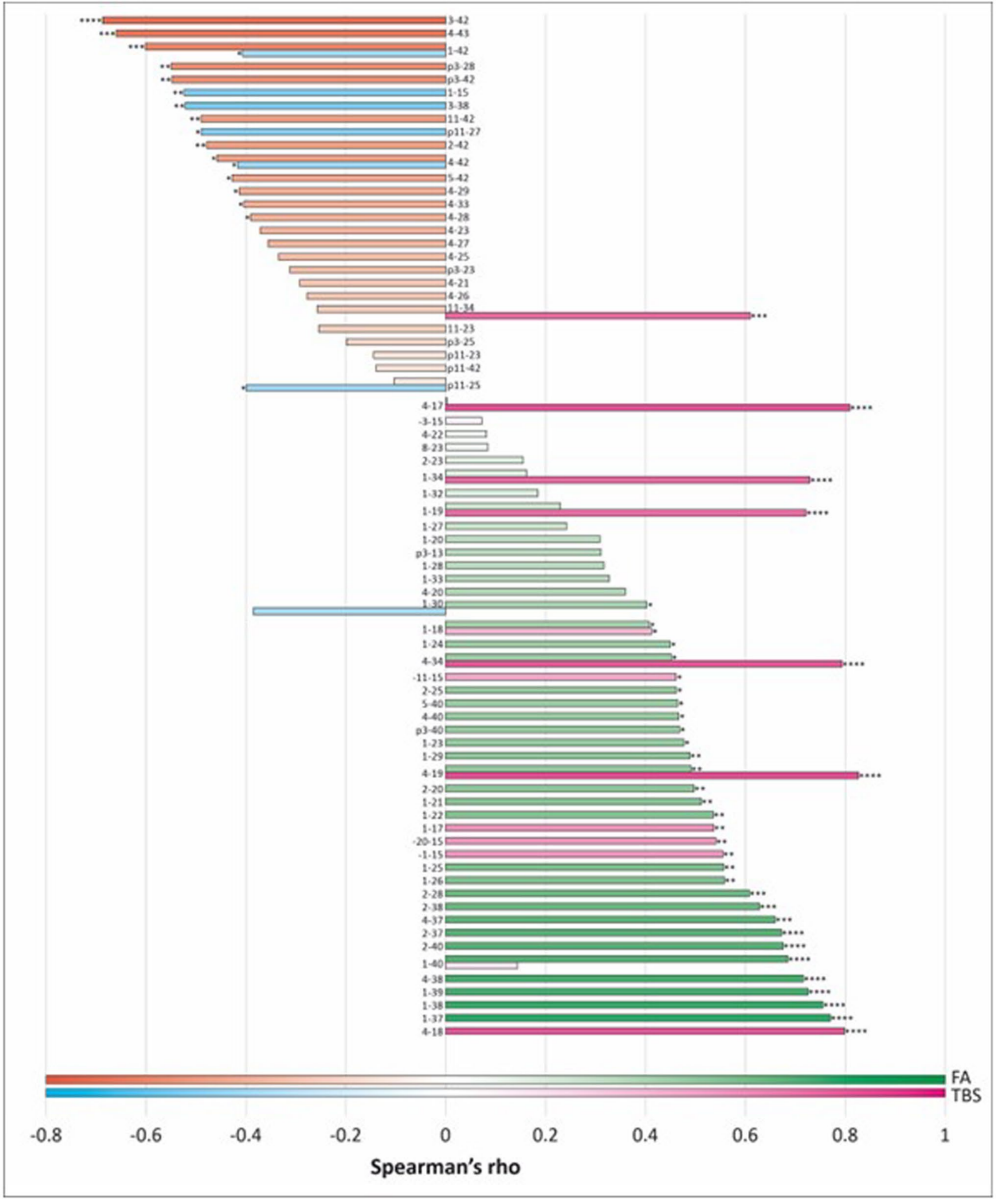
Correlation between the most abundant Aβ peptides and the CAA score in TBS and FA fractions. Bar lengths and color intensity represent intensity of correlations of the single Aβ peptide and the CAA score. The TBS fraction is colour-coded in pink for positive correlations and light blue for negative correlation. The FA fraction is represented in green for positive correlations and orange for negative correlations. Correlations were assessed using Spearman rho non-parametric test. Statistically significant correlations are indicated (^a^*p* < 0.05, ^b^*p* < 0.01, ^c^*p* < 0.001, ^d^*p* < 0.0001)

In search for a specific Aβ peptide signature for CAA across groups, we looked at correlations between histological scores and the most abundant LC–MS Aβ peptides among patients. We used a CAA score based on histological assessments of Aβ deposits in the vascular system, ranging from 0 to 38 (Supplementary Table 2), reflecting CAA abundance in each case. Mann CAA grading as in [51] of *APP*dup and DS-AD confirmed that cases with the lowest CAA scores corresponded to CAA grade 1 while the highest CAA scores corresponded to CAA grade 3 or 4. As shown in Fig. 8, in the TBS fraction, the Aβ peptides 4–34, 4–17, 4–19, and 4–18 were the ones showing the highest positive correlation with the CAA score, while Aβ1–42, 4–42, 1–30, and pE11-25 peptides were anti-correlated with the CAA score. In the FA fraction, peptides N-terminally truncated x–37, x–38, x–39, and x–40 were the ones showing the highest positive correlation with the CAA score, while peptides ending at amino acid 42 and 43, and pyroglutamylated forms showed significant negative correlations. Interestingly, the x–42 peptides showed significant negative correlations both in TBS and FA fractions, while a significant correlation was found for Aβ1-40 only in the FA fraction, suggesting that Aβ1-40 might not be similarly processed in the soluble and insoluble fractions. Of note, opposite correlations were found also for the 11–34 and 1–30 peptides; however, the negative correlations of 11–34 in FA and 1–30 in TBS were not significant.

### MALDI-TOF–MS imaging of *postmortem APP*dup cortical sections shows different Aβ profiles between parenchyma and blood vessels

**Fig. 9:**
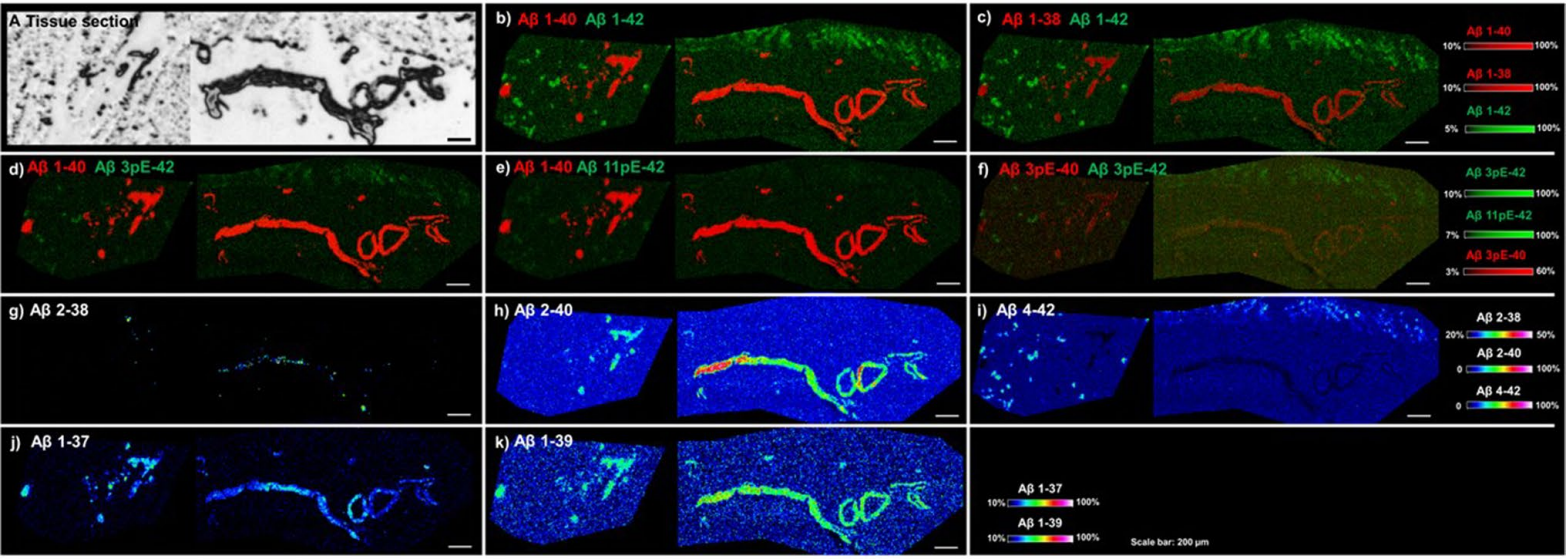
MALDI-TOF–MS representative ion images of Aβ peptides in *postmortem* brain of an *APP*dup case. MALDI ion images generated by visualizing the intensity distribution of individual ion signals (m/z) over the tissue array. (**a**) An optical image of brain sections. (**b–f**) MALDI overlay images of Aβ1-38, Aβx-40 and Aβx-42 peptides and (**g–k**) single ion images of different Aβ peptides revealing pathology specific chemical localization patterns

Since brain homogenates contain both parenchymal and vascular Aβ peptides, MALDI imaging was applied to one *APP*dup case having high CAA pathology (case #2) in order to obtain spatial Aβ signature. Here, the *postmortem* cortical brain tissue was analyzed in regions showing both parenchymal bloods vessels and leptomeningeal vessels (Fig. 9). The Aβ1–40 signal was mainly localized in arteries and leptomeningeal vessels while Aβ1–42 was detected exclusively in the parenchyma, showing no overlaps between the two peptides. We then analyzed Aβ peptides that were high in brain homogenates from *APP*dup (Aβ1-37, 1–38, 1–39, 2–38, and 2–40) and found them all localized exclusively in vessel walls both in the parenchyma and in the leptomeninges. The same applied to the respective pyroglutamylated forms of the x-40 peptides that localized to vessels while x–42 species localized to parenchymal plaques.

## Discussion

Cerebral amyloid angiopathy (CAA) is characterized by the accumulation of Aβ deposits in the walls of cerebral blood vessels, leading to loss of medial smooth muscle cells resulting in fragility of brain blood vessels and increasing the risk of intracerebral hemorrhage (ICH), though the exact cause of CAA is not fully understood [28]. Aβ peptides are also key components of the plaques found in the brain parenchyma of individuals with AD (sporadic, familial, as well as in DS). There is some overlap between the two conditions, and it has been suggested that CAA might contribute to the pathogenesis of AD by affecting perivascular clearance of Aβ [72].

To bring new elements in the discussion, we investigated the pattern of Aβ deposition in the parenchyma (plaques) and in blood vessels (CAA) and characterized Aβ species from *postmortem* brains of patients overexpressing the *APP* gene, thus overproducing Aβ peptides (*APP*dup, DS, and DS-AD) and compared them to patients with sAD or others carrying missense mutations in the *APP* gene (*APP*V717L and *APP*V717I) and controls. Interestingly, we previously observed that patients with DS-AD and *APP*dup had more severe CAA compared to sAD and *APP* mutations at codons 717 and 692 [51]. Here we could collect 7 *APP*dup brain samples from 6 different families while in Mann et al*.* samples came from 2 families [51].

To establish these comparisons, we performed immunohistochemistry on 49 fixed *postmortem* brain tissues (cortical regions) and IP-MS on 30 brain homogenates prepared from frozen tissue from the same cases (cortical and hippocampal regions), thanks to the availability of those rare cases in various European brain banks.

Scoring of Aβ deposits in the brain parenchyma and the vessel walls (arteries, arterioles, and venules) using 13 features (see Supplementary Table 2) showed that Aβ deposits were most prominent in the parenchyma in sAD and ADAD with *APP*V717L or *APP*V717I (twice the amount as compared with blood vessels). However, the ratio of Aβ deposits in parenchyma-to-blood vessels was 0.9 in DS-AD while it was inverted in *APP*dup with more than twofold more Aβ deposits in blood vessels compared with parenchyma. Interestingly Aβ deposits in the blood vessels were absent in the four cases with DS while deposits in the parenchyma were present. One might infer that a specific quantity of parenchymal Aβ contributes to heightened Aβ clearance and deposition within blood vessels. However, in cases involving *APP*dup, wherein Aβ deposits in the parenchyma were comparable to DS cases, there was also an elevated occurrence of Aβ deposits in blood vessels while among the four DS cases examined, Aβ deposits in blood vessels were entirely absent. This contrast suggests a potential protective mechanism in individuals with DS against Aβ deposition in blood vessels, keeping with the observation that rates of complications of CAA such as intracerebral hemorrhage may be less common in DS [9]. This safeguarding effect could arise from an enhanced blood–brain barrier permeability, alterations in the vascular unit favoring clearance, or a heightened local degradation of Aβ peptides in this compartment within DS individuals, resulting in a diminished inflammatory response in vascular smooth muscle cells and less Aβ deposits [80]. A recent population-based cohort study in individuals with DS using UK electronic health records showed increased risk of dementia in this population but decreased risk of hypertension and hypercholesterolemia [3]. It is also well known that individuals with DS show minimal atherosclerosis, including in their brain [34, 84]. Altogether these studies highlight a striking difference in the vascular unit of individuals with DS. One limitation of our study is that we solely conducted immunostaining on frontal cortical sections while the occipital lobe was found slightly more impacted by CAA in sAD (96% of cases) as compared to the frontal lobe (93% of cases) [81]. It will thus be important to analyze sections from different regions of the cortex including the occipital lobe to definitively exclude the presence of CAA in DS cases without AD. Additionally, the amount of Aβ deposits in the capillaries as well as the number of capillaries affected were higher in *APP*dup compared to DS-AD indicating a higher grading of CAA in *APP*dup, as observed previously [51]. In individuals with DS, Aβ peptides deposition along the arterial tree, arterioles, and finally capillaries would be less effective thus protecting this population from severe CAA and potentially ICH, as seen in patients with *APP*dup [10, 27, 64].

In parallel to the evaluation of 13 features related to Aβ deposition in the parenchyma and in blood vessels, we classified CAA according to Mann et al*.* using the defined four grades [51] (data not shown). We found that 6/7 *APP*dup cases were Mann CAA grade 3 while the remaining case (#7 in Fig. 3) was Mann CAA grade 1 with few faint plaques, low CAA in the cortex but higher in meninges and no Aβ deposits in the capillaries. Here we examined three cases with *APP*dup (#1, #2 and #3) already analyzed in the study of Mann et al*.* as cases #1, #3, and #2, respectively [51]. In comparison, we obtained similar Mann CAA grading for two cases while one case graded 3 here and 2 in Mann et al*.* (our case #3) with few Aβ plaques, medium CAA and few Aβ positive capillaries.

The role of the genetic AD risk factor *APOE* ε4 in CAA has been clearly established and especially the higher frequency of Aβ deposits in the capillaries in sAD *APOE ε4* carriers [73]. However, here we had only one *APP*dup and two DS-AD cases carrying one *APOE ε4* allele (cases #7 and cases #3 and #5, respectively, Fig. 3 and Supplementary Table 1) in which both CAA and Aβ deposits in the capillaries were not worse than in cases homozygous for the *APOE ε3* allele, which is consistent with the fact that *APP* duplication is a much stronger genetic determinant of CAA than the *APOE ε4* allele.

Contrary to differences in Aβ deposition observed between pathological groups, amounts of phosphorylated tau (pTau) deposits only showed minor differences across the groups of AD cases (sAD, DS-AD, *APP*dup, and *APP* missense mutations V717L and V717I). We scored phosphorylated pathological tau (pTau) as well as, more specifically, the numbers of neuritic plaques, neurofibrillary tangles, and neuropil threads. Cases with DS-AD showed the highest scores for pTau, neuritic plaques and neurofibrillary tangles while those scores were lower in *APP*dup suggesting the involvement of additional mechanisms beyond *APP* overexpression on tau pathology in DS. Indeed, genes from human chromosome 21 other than *APP* have been shown to increase amyloid and tau pathologies [55, 68]. Moreover, the *DYRK1A* gene mapping to human chromosome 21, encoding a serine/threonine kinase, has been shown to increase both tau expression and tau phosphorylation [47, 59].

Our second aim was to investigate the same cohort focusing on the Aβ peptide patterns. Brain samples were homogenized and fractionated into TBS (water soluble) and FA (water insoluble) fractions, and immunoprecipitated using a mix of 4G8 and 6E10 monoclonal antibodies targeting Aβ species which were then identified using mass spectrometry. This allowed for the analysis of a large variety of Aβ species, including peptides truncated both N- and C-terminally, Aβ peptides extended N-terminally of the BACE1 cleavage site, as well as peptides post-translationally modified by pyroglutamate formation. For the MALDI analysis, FA fractions of matching cortical and hippocampal frozen tissue were used. The analysis revealed a correspondence of Aβ species and relative amount between the two regions in the different groups. A more in-depth quantitative analysis of the cortex extracts, in both TBS and FA fractions, was performed with LC–MS. The amounts of peptides in the soluble TBS fraction were highest in DS-AD and in *APP*dup samples due to the overexpression of the *APP* gene while they were comparable in the insoluble fractions for all samples from patients with AD.

When analyzing more in detail the insoluble fractions containing Aβ species present in deposits, the *APP*dup and DS-AD groups showed the highest levels of Aβx–40 peptides (Fig. 7b). Of note, levels of Aβx–40 peptides were high in the DS-AD group and very low in the DS group. On the other hand, the levels of Aβx–42 peptides were somewhat higher in the DS compared to the DS-AD group (Fig. 7b, d). This might point towards changes in APP processing during the progression of the disease. Our study also revealed a specific profile in DS-AD samples which were identified with the highest levels of pyroglutamylated forms Aβ3pE–40/–42 and Aβ11pE–40/–42, as shown previously [25, 32, 33]. The enzyme responsible for modifying Aβ peptides into the pyroglutamylated form, glutaminyl cyclase, may be more active in individuals with DS, although the underlying reasons for this heightened activity remain unknown [17, 66]. Therefore, treatments that reduce pyroglutamylated peptides may have potential in treatment of individuals with DS [4, 60, 69].

In brain samples with *APP*dup, we found very high amounts of Aβ1–34 and Aβ1–38, and their respective truncated forms at the N-terminus at positions 2- and 4-, and the same was observed with Aβ peptides ending at 37 and 39, although to a lesser extent. Previous studies showed higher abundance of Aβ1–40 and Aβ2–40 in the brain of AD patients with CAA as well as Aβ1-38 while in brains without CAA the most abundant species were 1–42, Aβ2–42, Aβ3pE–42 and Aβ11pE–42 [24, 34, 65]. Having here analyzed for the first-time brain samples with massive CAA in cases involving *APP*dup, we successfully obtained a more thorough profile by including specific species, notably the Aβ2–x and Aβ4–x peptides. The enzyme meprin-β has been demonstrated to be able to cleave Aβ peptides at position 2 [65, 67]. Whether the level and/or activity of meprin-β are increased in *APP*dup remains unknown.

The distinctive patterns of Aβ peptides identified in brain homogenates from *APP*dup and DS-AD suggest a potential association with vascular Aβ deposits. However, given that brain homogenates encompass both parenchymal and vascular Aβ, discerning the precise origin becomes challenging. To unravel the spatial distribution of Aβ species, we employed MS imaging on an *APP*dup brain sample. Our findings revealed that Aβ1–37, Aβ1–38, Aβ1–39, and Aβ1–40 are localized in blood vessels and leptomeningeal vessels, while Aβ1–42 is detected solely in the parenchyma thus confirming results obtained in sAD [38]. The same distinct spatial distribution applies to N-truncated peptides. These results strongly suggest that these specific Aβ species are selectively deposited in vessel walls, indicating clearance or local production, as discussed above. However, this finding will require replication in future studies on additional cases with sAD and DS-AD.

Finally, through the correlation of the quantified levels of all Aβ peptides with a CAA score that indicates CAA abundance and distribution across all analyzed cases, we validated that Aβ1–37, Aβ1–38, Aβ1–39, and Aβ1–40, along with their N-truncated forms, exhibited the highest correlations. Conversely, there was an inverse relationship between CAA score and Aβ1–42, Aβ1–43, Aβ3pE–x, as well as Aβ11pE–x.

In conclusion, we have identified a distinctive pattern of Aβ peptides that shows correlation with CAA. This discovery was made possible through the application of complementary methods such as immunohistochemistry, MALDI-TOF–MS and LC–MS on brain homogenates and MALDI-TOF–MS imaging on brain sections.

Aβ1–37, Aβ1–38, Aβ1–39, and Aβ1–40, along with their N-truncated forms exhibited the strongest association with CAA (Fig. 8), with the Aβ1-x showing the highest amounts (Fig. 7). It will be important to delve into whether these peptides are selectively generated or degraded within the vascular unit, and whether some might be useful as biomarkers for CAA in CSF or plasma. Decreases of Aβ1–34, Aβ1–37, Aβ1–38, Aβ1–38 and Aβ1–40 but not Aβ1–42 have already been identified in the CSF of non-AD patients with CAA [76].

Our research further confirms that APP overexpression is a very strong genetic determinant for CAA, with a particularly pronounced effect in ADAD cases carrying *APP* duplication compared to DS. This contrast provides insights into potential protective mechanisms within the DS population, thereby supporting the inclusion of individuals with DS in clinical trials for anti-Aβ immunotherapy.

### Supplementary Information

Below is the link to the electronic supplementary material.

Supplementary file1 (XLSX 22 kb)

Supplementary file2 (XLSX 22 kb)

Supplementary file3 (XLSX 23 kb)

## Data Availability

All data are available upon request.
